# Mapping the Distribution of *Curculio davidi* Fairmaire 1878 under Climate Change via Geographical Data and the MaxEnt Model (CMIP6)

**DOI:** 10.3390/insects15080583

**Published:** 2024-07-31

**Authors:** Junhao Wu, Xinju Wei, Zhuoyuan Wang, Yaqin Peng, Biyu Liu, Zhihang Zhuo

**Affiliations:** College of Life Science, China West Normal University, Nanchong 637002, China; wujunhao0824@gmail.com (J.W.); weixinjuxx@foxmail.com (X.W.); zhuoyuan0620@163.com (Z.W.); pengyaqin2023@foxmail.com (Y.P.); biyuliuql@foxmail.com (B.L.)

**Keywords:** *Curculio davidi*, MaxEnt model, climate change, potential distribution

## Abstract

**Simple Summary:**

*Curculio davidi* (Curculionidae, Coleoptera) is distributed throughout all chestnut-producing regions in China. Its hosts include almost all species of the genus *Castanea*, as well as hazel and oak trees. The weevil primarily damages chestnuts during its larval stage, with infestation rates exceeding 80%. It poses a significant threat to the production and storage of chestnuts. In this study, the MaxEnt model (version 3.3.4) and ArcGIS software (version 10.8) were used to map the current and future suitable habitat distribution of *C. davidi* under climate change, considering known geographical data and environmental factors. The results indicated that under current climate conditions, the high-suitability areas of *C. davidi* are predominantly concentrated in the middle and lower reaches of the Yangtze River Plain and eastern Sichuan in China. Predictions based on future climate scenarios showed a reduction in the area of high- and low-suitability zones for *C. davidi*, with only moderate suitability zones expanding in size. This study provides data support for the management strategies of *C. davidi* and offers new insights into the dynamic changes of similar forestry pests.

**Abstract:**

*Curculio davidi* is a major pest in chestnut-producing regions in China, and there have been many studies on its occurrence, biological characteristics, and management strategies. However, few of them have focused on the distribution changes of the pest under climate change. In this study, the MaxEnt model (version 3.3.4) and ArcGIS software (version 10.8) were first employed to map the current and future (2050 s and 2080 s) suitable habitat distribution of the weevil under climate change (CMIP 6: SSP1-2.6, SSP2-4.5, and SSP5-8.5). The results indicate that the highly suitable areas for *C. davidi* are mainly concentrated in Hubei, Henan, Anhui, Jiangxi, Jiangsu, Zhejiang, the coastal areas of Shandong, and eastern Guizhou, northwestern Hunan, and northeastern Sichuan provinces in China. Through the Jackknife test of 19 climate factors, six climate factors affecting the distribution of *C. davidi* were identified, with precipitation from July (Prec7), precipitation of warmest quarter (Bio18), and temperature seasonality (standard deviation × 100) (Bio4) contributing a combined percentage of 86.3%. Under three different climate scenarios (CMIP 6: SSP1-2.6, SSP2-4.5, and SSP5-8.5), the area of moderately suitable regions is projected to increase by 22.12–27.33% in the 2050 s and by 17.80–38.22% in the 2080 s compared to the current distribution, while the area of highly suitable regions shows a shrinking trend. This study provides data support for the management strategies of *C. davidi* and offers new insights into the dynamic changes of similar forestry pests.

## 1. Introduction

**Figure 1:**
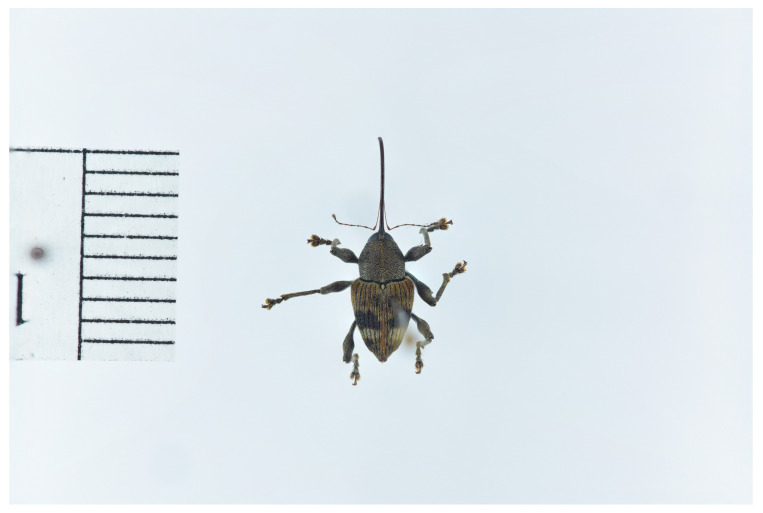
Adult of *C. davidi*.

*Castanea mollissima* Blume 1851 is a nut with high nutritional value, originally from China. It has since been distributed to various regions around the world, including Asia, Europe, North America, Africa, and others [1]. In China, this plant mainly grows in mountainous areas at altitudes ranging from 370 to 2800 m. However, *Curculio davidi* Fairmaire 1878 [2] (Figure 1) is found in all the chestnut-producing regions of China. The pest can attack a wide range of host plants, including most species of the *Castanea* genus, as well as hazel and oak trees. This weevil primarily causes damage to chestnuts during its larval stage, with infestation rates often exceeding 80%. As a result, it poses a significant threat to chestnut production and storage.

**Figure 2:**
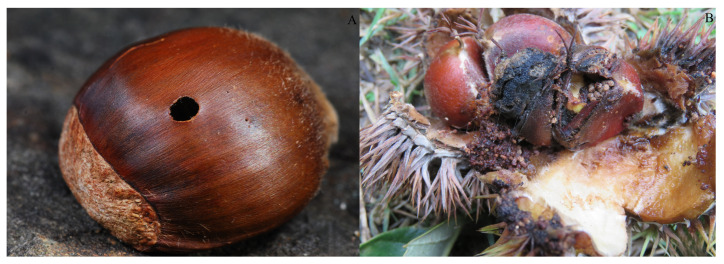
Holes in the nutshell of chestnuts. (**A**): the site of egg laying; (**B**): Symptoms of *C. davidi* infestation.

In recent years, there have been many studies on *C. davidi*, especially with respect to its biological characteristics and integrated control. For example, Higaki and Toyama’s research indicates that some larvae of *C. davidi* can complete pupation after just one winter [3]. After hatching, larvae of *C. davidi* begin feeding on the kernels of chestnuts. During feeding, they create pits inside the chestnuts, which gradually deepen and widen as the larvae grow. These pits are filled with insect droppings, and the infested chestnuts partially or completely lose their germination capacity and edible value [4]. This is the main symptom of the pests harming host fruits (Figure 2). In addition, most of the time, the larvae live inside the fruit, and they will immediately enter the soil to complete pupation and eclosion when they leave the host fruit. All these characteristics make the control of *C. davidi* more difficult [5]. Therefore, we should base our approach on the leading idea of prevention and the principles of safety, effectiveness, economy, and simplicity to manage the pest. If *C. davidi* poses a serious threat to the host, integrated management (chemical, biological, and physical control) should also be employed [6]. There have been many studies on its occurrence, biological characteristics, and management strategies [7], but few have focused on the distribution changes of the pest under climate change.

The occurrence of *C. davidi* and the climate in nearby woodlands are related. The link between climate change and species distribution has been a research highlight. Forecasting changes in the suitable living areas of the pest under climate change is crucial for understanding the development pattern of pests and establishing a comprehensive pest prevention and control system [8]. To address these problems, various species distribution modeling (SDM) approaches, also known as ecological niche modeling (ENM), have been developed since the 1980s [9]. SDMs are constructed using known distribution data for species and relevant environmental variables, following specific mathematical algorithms. The model visualizes prediction results in corresponding time and space for data representation. These results are used to analyze the ecological requirements of the species [10]. In recent years, several techniques have been used to model the ecological niches and geographic distribution of species. Various ecological niche models, such as MaxEnt, GARP, Bioclim, etc., have been widely used in biogeography, conservation biology, and ecology research and have shown good results in predicting the potential geographic distribution of species. However, systematic biological survey data for most regions is often limited or sparse [11]. MaxEnt considers only the “occurrence” (presence only) of the target species in the region and integrates the maximum entropy of species occurrence for a given environmental factor to analyze the fitness of the species in the predicted area. It is a general model that uses known partial information to make predictions or inferences about unknown messages [11]. Therefore, the MaxEnt model requires fewer data to make predictions and is more accurate than other ecological niche models [12]. Due to these advantages and its ease of use, the MaxEnt model is the most widely used SDM algorithm [13]. Furthermore, due to ArcGIS’s excellent visualization, it is often adopted in conjunction with MaxEnt to analyze the potential habitable areas of species [14,15,16].

In this study, both the MaxEnt model (version 3.3.4) and ArcGIS software (version 10.8) were first used to predict the habitability of *C. davidi* in China and to determine the changes in the distribution area in different periods. The results will reveal the significant impact of climate change on the distribution of the pest and provide a scientific and theoretical basis for the development of reasonable management strategies.

## 2. Materials and Methods

### 2.1. Species Distribution Data

**Figure 3:**
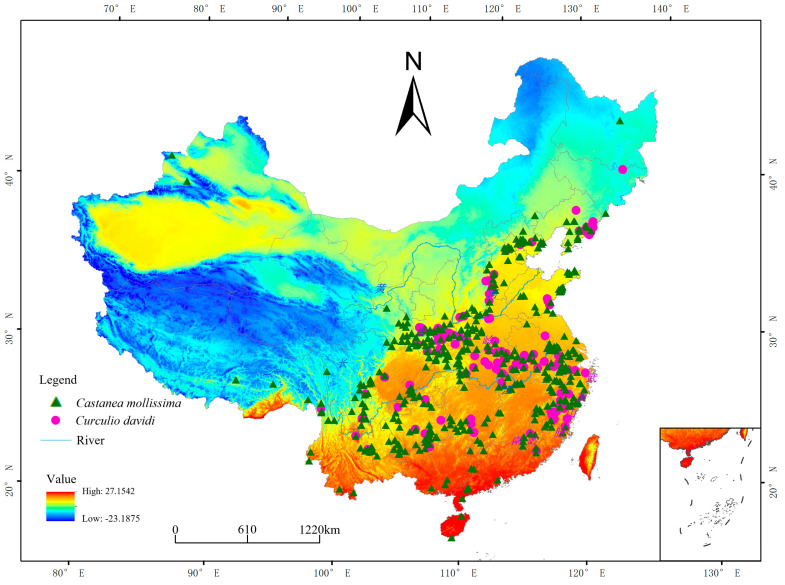
Geographical distribution points of *C. davidi* and *C. mollissima* in China. Pink points: *C. davidi*; green triangle: *C. mollissima*; dark blue: average annual low temperature; positive red: average annual high temperature.

There are four main methods for obtaining the geographic distribution of species: field surveys, reviewing literature, querying databases, and referring to the specimen museum. The distribution data of *C. davidi* and *C. mollissima* were mainly obtained from the Global Biodiversity Information Facility (GBIF, https://www.gbif.org/, accessed on 17 July 2024). The obtained distribution points were carefully checked and screened, resulting in a total of 132 distribution points (Figure 3). The longitude and latitude of these distribution points were determined using Google Maps. The actual distribution points of *C. davidi* were then stored as “CSV” files, following MaxEnt’s requirements, in the order of species name, distribution point longitude, and distribution point latitude.

### 2.2. Environmental Variables

Environmental variables affecting species distributions differ at various spatial scales. At relatively large scales, species interactions are often attenuated, and climatic variables play a major role. [17]. We used 19 bioclimatic variables as variables involved in the study, with environmental variables derived from the World Climate Database (http://www.worldclim.org/, accessed on 27 May 2024) composed of global climate records for the years 1970–2000. Because there is a large variation in the effect values of environmental variables, the Pearson correlation coefficient analysis was conducted in SPSS 22 to identify the environmental variables that contribute most to the model. The objective was to eliminate the impact of covariance on the modeling process and the interpretation of the results [18]. It is considered that variables with correlation coefficients greater than or equal to 0.8 and high contribution rates are factors that remove the influence of covariance on the modeling process and results. In conclusion, we retained six variables based on the bioclimatic characteristics of *C. davidi* and the contribution of the variables: July precipitation (prec7), warmest quarter precipitation (bio18); standard deviation of seasonal temperature variation (bio4); coefficient of variation of precipitation (bio15); warmest quarter mean temperature (bio10); and May precipitation (prec5). These six variables were used to model the MaxEnt of *C. davidi’s* distribution in China.

For future climate data, the Beijing Climate Center Climate System Model 2 Medium Resolution (BCC-CSM2-MR) in CMIP6 was selected, and three shared socioeconomic pathways (SSPs) in CMIP6 (SSP1-2.6, SSP2-4.5, and SSP5-8.5) were adopted to predict the potential distribution areas of *C. davidi*. The SSP1-2.6 represents a minimum GHG emission scenario, assuming that CO_2_ concentration will reach 450 × 10^−6^ L·L^−1^ in 2100 and the that global average temperature will increase by 0.2 to 1.8 °C. The SSP2-4.5 represents a medium GHG emission scenario, assuming a CO_2_ concentration of 650 × 10^−6^ L·L^−1^ in 2100 and a global average temperature increase of 1.0 to 2.6 °C. The SSP5-8.5 represents a maximum GHG emission scenario, assuming a CO_2_ concentration of 1350 × 10^−6^ L·L^−1^ and a global average temperature increase of 2.6 to 4.8 °C [19].

### 2.3. Modelling Process

The ROC curves were employed to assess the accuracy of the MaxEnt model. The curve is currently the most effective metric widely used to verify the accuracy of simulation results for model prediction. The AUC values represent the area enclosed by the ROC curve and the horizontal coordinate. The average AUC values range from 0.5 to 1.0, and the closer the AUC value is to 1, the stronger the correlation between the environmental variables and the predicted species geographic distribution model, indicating higher prediction accuracy. The natural distribution points of *C. davidi* and environmental variables were imported into MaxEnt software (version 3.3.4), and then 75% of the natural distribution points were randomly selected as the training set of the model and the remaining 25% as the test set. The results were output in logistic format to obtain the occurrence index for each raster. We performed twenty bootstrap replications of the model and used the average index as the final model result.

The default settings of the MaxEnt model are currently based on extensive empirical tuning studies. However, there is always some degree of spatial or sampling bias at the point of occurrence [20]. To minimize this bias, a bias file can be generated using species occurrence data and the environment to estimate and develop a two-dimensional kernel density raster. This bias file can then be used in MaxEnt distribution modeling. The “ENMeval” package was used to develop the bias file for all MaxEnt models, which increased the rigor of the modeling process [21,22].

The Jackknife test was chosen to analyze the environmental impact factor. This test provides a plot that shows the importance of climate factors in this context. The test plots revealed multiple bands, with each band representing the gain values from regularization training using only a single climate factor to construct the species distribution fit model. The length of the strip indicates the training gain value, with longer strips indicating a higher contribution of the climate factor to the species distribution [23].

### 2.4. Classification of Suitable Grades

In order to classify suitable grades, the *.asc format was converted to the raster format using the conversion tools of ArcGIS software (version 10.8). Chinese administrative regions were overlaid at a scale of 1:1000 km. The suitable areas were then categorized into four classes using Jenks’ natural break classification method. These classes are as follows: *p* < 0.05, indicating unsuitable areas; 0.05 ≤ *p* < 0.33, indicating low fitness areas; 0.33 ≤ *p* < 0.66, indicating moderate fitness areas; and *p* ≥ 0.66, indicating high fitness areas.

## 3. Results

### 3.1. Model Result Verification

**Figure 4:**
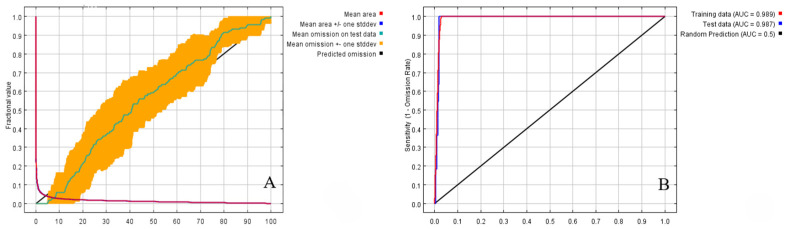
The accuracy of the MaxEnt model on *C. davidi*. (**A**): Curve of omission and predicted area; (**B**): ROC curve of potential distribution prediction.

The omission rate provides information on model differences and overfitting, evaluating the data used at a specific threshold. If the test omission rate is consistent with the theoretical omission rate, the model is proven to be well constructed. To demonstrate the spatial autocorrelation of modeled data, the test omission rate should be either higher or lower than the theoretical omission rate [24]. According to the output omission rate curve of the MaxEnt model after 10 repetitions of the mean value (Figure 4A), it can be observed that the predicted omission rate of the sample was in high agreement with the omission rate of the test sample, indicating that the model was well constructed and there was no spatial autocorrelation in the modeled data.

The accuracy of the MaxEnt model is evaluated by plotting the ROC curve in MaxEnt software to calculate the AUC value. As shown in Figure 4B, *C. davidi* achieved an AUC value of 0.988. Therefore, the accuracy of the model in this study was rated as “excellent”. The test results showed that the model can provide reliable information on the effect of climate change on the distribution of *C. davidi*.

The percentage contribution and cumulative percentage contribution of the six climate factors affecting the distribution of *C. davidi* were determined using the Jackknife test as shown in Table 1. They were prec7 (42.3%), bio18 (22.3%), bio4 (21.7%), bio15 (7.2%), bio10 (4.1%), and prec5 (2.4%). The cumulative contribution of these six climatic factors reached 100%, and it could be considered that these six climatic factors were the key dominant factors affecting the distribution of *C. davidi*.

### 3.2. Current Distribution Forecast

**Figure 5:**
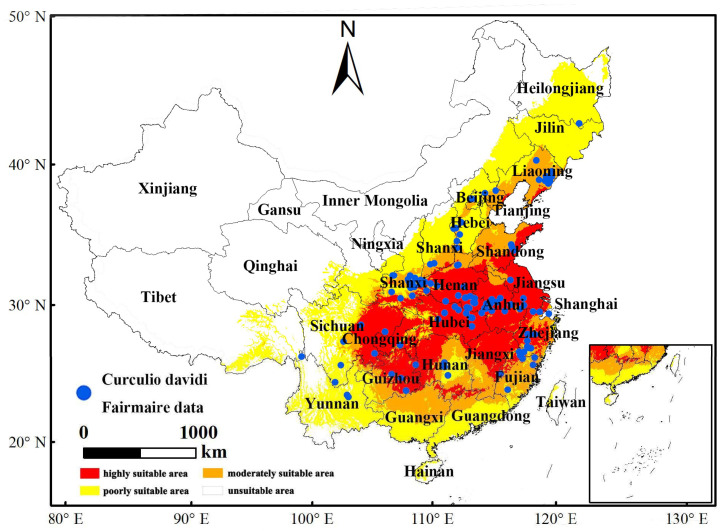
Current suitable climatic distribution of *C. davidi* in China. Red: highly suitable area with a probability of higher than 0.66; orange: moderately suitable area with a probability of 0.33–0.66; yellow: poorly suitable area with a probability ranging from 0.05–0.33; White: unsuitable areas.

Employing the ArcGIS software and the MaxEnt model, the habitat distribution of *C. davidi* was obtained (Figure 5). The red represents highly suitable area, mainly concentrated in Hubei Province, Henan Province, Anhui Province, Jiangxi Province, Jiangsu Province, Zhejiang Province, coastal areas of Shandong Province, eastern Guizhou Province, northwestern Hunan Province, and northeastern Sichuan Province in China. These places experience more serious infestations of *C. davidi*. As shown in Table 2, the current total area of highly suitable habitat for *C. davidi* is 65,251 km^2^, accounting for 0.68% of the total area of China. Hubei province has the largest highly suitable area, with 8426 km^2^, accounting for 4.53% of the province’s total area and 12.91% of the country’s highly suitable area. These findings indicate that the areas where *C. davidi* is highly adapted are primarily concentrated in the southwestern and eastern parts of the country.

### 3.3. Environmental Variable Analysis

**Figure 6:**
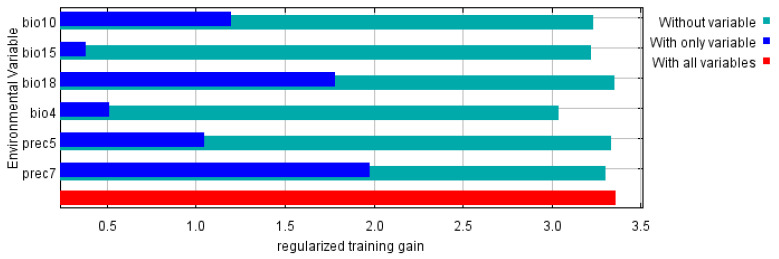
Importance of environmental variables to *C. davidi* via Jackknife test.

This study performed a Jackknife analysis of the six environmental variables that significantly influenced *C. davidi*, and the results are shown in Figure 6. The six main impact factors were prec7 (1.96), bio18 (1.74), bio10 (1.08), prec5 (0.91), bio4 (0.31), and bio15 (0.16). This suggested that temperature and precipitation associated with climate change were going to be the main determinants affecting the distribution of *C. davidi*.

**Figure 7:**
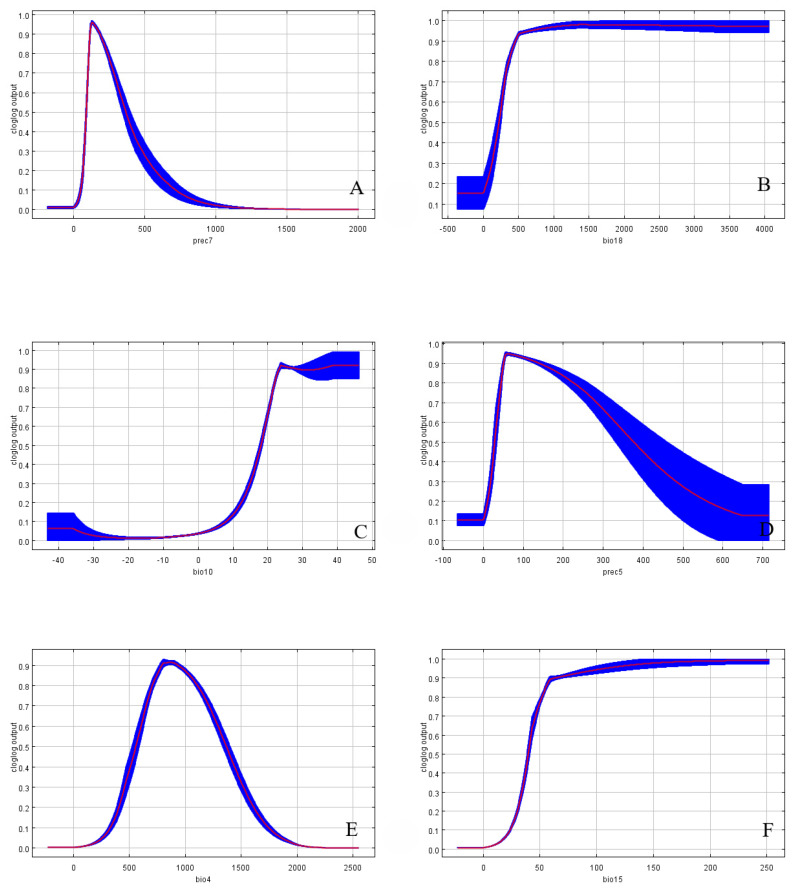
Response curves of the environmental variables that contributed most to the MaxEnt models. (**A**): Precipitation of July (prec 7); (**B**): Precipitation of Warmest Quarter (bio 18); (**C**): Mean Temperature of Warmest Quarter (bio 10); (**D**): Precipitation of May (prec 5); (**E**): Temperature Seasonality (Standard Deviation of × 100) (bio 4); (**F**): Precipitation Seasonality (Coefficient of Variation) (bio 15).

Based on the response curves of the *C. davidi* probability distribution depicted in Figure 7, the ranges of future distribution variables have been determined. The Prec7 (Figure 7A) has a precipitation range of 107.22–324.13 mm, with an optimal precipitation of 103.13 mm. The curve shows a steep rise and fall, indicating that precipitation in July is a major variable influencing the distribution of *C. davidi*. The Bio18 (Figure 7B) has a suitable range of 305.02–1498.07 mm, with an optimal value of 409.35 mm. Unlike prec7, this variable shows a rapid increase followed by a gradual decline. It is noteworthy that bio10 (Figure 7C) exhibits two suitable ranges within the selected temperature range: the first rises from 0 at −20 °C, peaks at 23.58 °C, then declines slowly to 29.90 °C, followed by a slow rise to 39.95 °C and stabilization. Throughout this change, two temperature intervals are considered suitable: 20.55–23.58 °C and 29.90–39.95 °C, with peaks at 23.58 °C and 39.95 °C corresponding to suitability values of 0.92. The Prec5 (Figure 7D) has a suitable range of 44.08–299.32 mm, with an optimal value of 55.15 mm. From Figure 5, it can be seen that the current highly suitable areas of *C. davidi* are mainly distributed in the eastern parts of the country, northern China, and the Sichuan Basin, influenced by the monsoon. The monsoon shows a “northward advance and southward retreat” pattern during the rainy season from June to September, resulting in less precipitation compared to July. The Bio4 (Figure 7E) has a suitable range of 674.2–1301.02 °C, with an optimal value of 798.1 °C. The curve shows a steep rise and fall, indicating that temperature fluctuation is a significant factor limiting the distribution of *C. davidi*. Bio15 (Figure 7F) has a suitable range of 45.68–250.36 mm, with an optimal value of 58.63 mm. From this range, it can be seen that the seasonal variation in precipitation in the highly suitable distribution areas of *C. davidi* is relatively large, further illustrating that precipitation is an important factor restricting the distribution of *C. davidi* (details in Table 3).

### 3.4. Future Distribution Forecast

**Figure 8:**
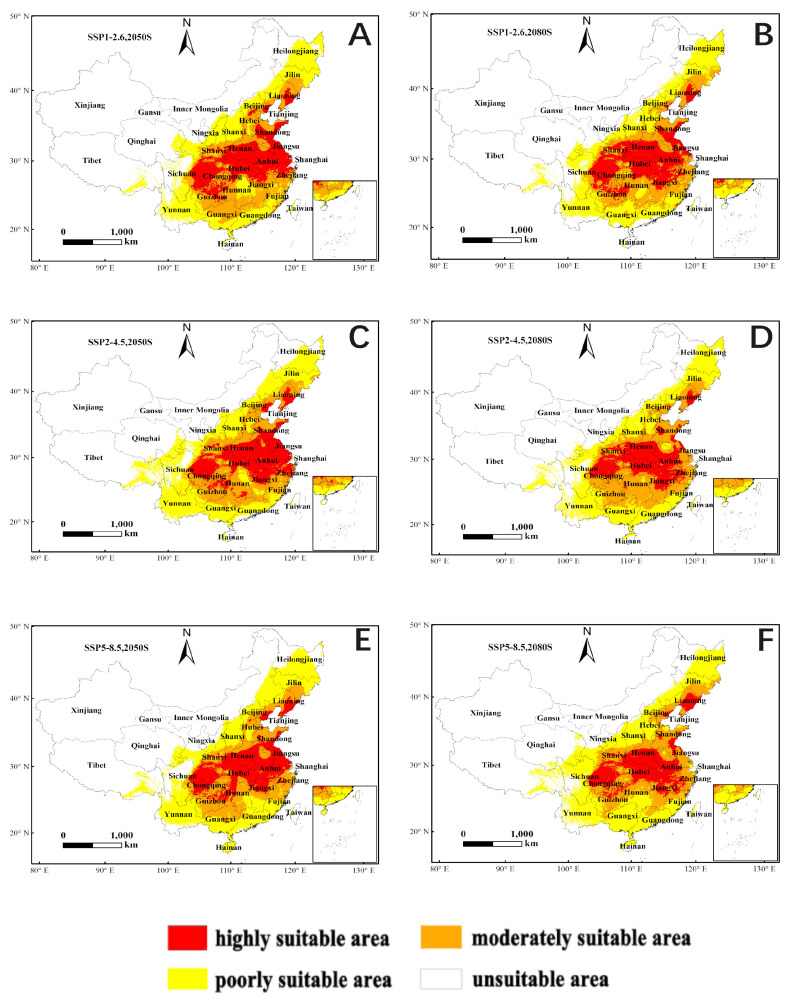
Appropriate potential distribution of *C. davidi* under different climate change scenarios in China. The color level of this area shows the probability of *C. davidi*, with red indicating that the area is highly suitable for a probability higher than 0.66, orange representing a moderately suitable area with a probability of 0.33–0.66, yellow indicating a poorly suitable area with a probability of 0.05–0.33, and white representing the unsuitable area response curves of the environmental variables that contributed most to the MaxEnt models. (**A**): SSP1-2.6 (2050S); (**B**): SSP1-2.6 (2080S); (**C**): SSP2-4.5 (2050S); (**D**): SSP2-4.5 (2080S); (**E**): SSP5-8.5 (2050S); (**F**): SSP5-8.5 (2080S).

**Figure 9:**
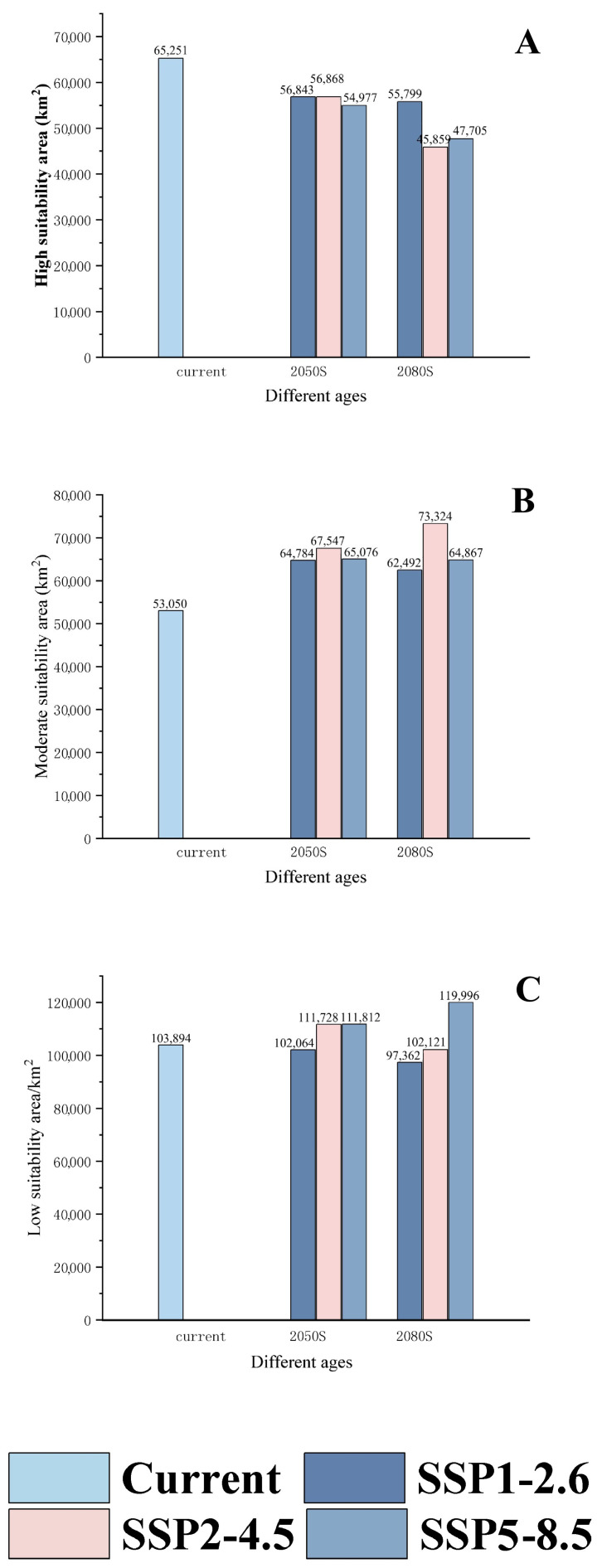
The area changes of suitable habitat for *C. davidi* under different climate change scenarios. (**A**): highly suitability area; (**B**): moderate suitability area; (**C**): low suitability area.

As shown in Figure 8, the distribution of *C. davidi* is depicted under three climate change scenarios (SSP1-2.6, SSP2-4.5, and SSP5-8.5) and two time periods (2050 s and 2080 s). Table 4 reveals that the area of moderately suitable habitat expanded to varying degrees in both the 2050 s (22.12%, 27.33%, 22.67%) and the 2080 s (17.80%, 38.22%, 22.28%) predicted results compared to the existing distribution under the three climate change scenarios. Conversely, the highly suitable areas experienced varying degrees of reduction compared to the existing distribution. Specifically, under the SSP2-4.5 climate change scenario, the 2080 s exhibited the largest increase in the area of moderately suitable habitat (38.22%) compared to the existing distribution, while simultaneously experiencing a maximum decrease of 29.27% in the area of highly suitable habitat. This decrease was the most significant when compared to the existing distribution. Notably, the percentage increase in the area of moderately suitable habitat under the SSP2-4.5 climate change scenario was the highest among the different climate change effects for both the 2050 s and 2080 s, as illustrated in Table 4 and Figure 8 and Figure 9.

## 4. Discussion

Previous research on *C. davidi* focused on its morphology, molecular biology, chemical control, and biological control [4,25,26,27], and few studies focused on its distribution. Those studies can only address a limited range of pests and cannot predict their future survival, making it difficult to prevent them proactively. Our study aims to simulate the potential distribution of *C. davidi* in China using the MaxEnt model. This model provides reliable data for understanding the future distribution of *C. davidi* and offers a more effective solution to the issues mentioned earlier. Unlike other similar models, the MaxEnt model is not hindered by limited information on species distribution, making it a preferred method in pest research. Additionally, the MaxEnt model demonstrates superior accuracy in predicting ecological niche distributions for various species [28]. Its efficient data processing capabilities make it particularly useful in predicting the large-scale potential distribution of species, offering broader prospects for ecological niche studies.

Based on the MaxEnt model’s prediction, the current distribution of *C. davidi* in China spans from 92.94° E to 123.92° E longitude and 17.89° N to 47.60° N latitude. The highly suitable areas are mainly found in the North China Plain, the middle and lower Yangtze River Plain, the Sichuan Basin, and the northern part of the Yunnan-Guizhou Plateau. Chestnuts, which are one of China’s oldest and earliest domesticated fruit trees, are predominantly grown in Hebei, Henan, Hubei, Shanxi, Yunnan, and other provinces. According to the regional distribution map of the Third National Agricultural Census conducted in 2021, 19% of the top 100 counties with chestnuts as the main cash crop are located in Shanxi Province, with an additional 10% in Yunnan Province. This overlaps with the highly suitable zone of our study for *C. davidi*. However, under different climate change scenarios, the highly suitable areas for *C. davidi* are expected to decrease, while the moderately suitable areas will expand. It is projected that highly suitable areas may transition to moderately suitable zones as a result of global warming when compared to the current distribution range (92.94° E to 123.92° E, 17.89° N to 47.60° N). As a result, it is crucial to determine appropriate ranges and implement protective measures for pest control in order to establish effective pest control regulations.

The occurrence, reproduction, and spread of pests are closely related to the environment, hosts, agroecosystems, and management levels. Meteorological elements are an important component of environmental variables. When other factors remain relatively fixed, meteorological factors can become decisive in determining the large-scale distribution of pests and diseases [29]. The percentage contribution of two climatic factors, prec7 and bio18, indicates that precipitation associated with climate change will be the main determinant affecting the occurrence of *C. davidi*, and this timing overlaps with the life history cycle of *C. davidi*. The pest completes its lifecycle in two years and overwinters as mature larvae in the soil layer. A batch of larvae pupates in the soil in mid-June, and after about 50 days, they emerge as adults in early August. The eggs hatch into larvae in about ten days, and the larval period lasts about twenty days. Therefore, in addition to climate variables, soil factors may also have an impact on its distribution.

The amount of rainfall can always affect the survival of terrestrial organisms. For example, in the case of adult *C. davidi*, if the amount of precipitation is too high, it may affect its foraging behavior. It has been shown that in the dry-slope chestnut production area of the Taihang Mountains, the amount of rainfall during the expansion period has a greater influence on the ripening period of chestnuts. The ripening period of chestnuts in the fruiting season (July and August) with more rainfall (above 450 mm) can be 10 days earlier than those with less rainfall (below 200 mm) and at least 6 days earlier. In short, the more rainfall in the warmest season, the earlier the ripening period of chestnuts and the better the harvest [30]. Two climatic factors, prec7 and bio18, coincide with the precipitation indicators in summer, and the presence of rainfall and *C. davidi* are positively correlated with the effect of rainfall on chestnut ripening during the warmest season when precipitation is the influencing factor, overlapping with the effect of rainfall on chestnut ripening. Thus, precipitation can indirectly affect the distribution of the pest by affecting the ripening period of host fruits. In other words, the host can also affect the distribution of *C. davidi*. In this work, we have marked the distribution points of *C. mollissima* and *C. davidi* in Figure 1, and the results show that the distribution of the pest is significantly correlated with the host, which is consistent with the above results.

Climate factors not only affect the distribution of hosts but also their nutritional composition. The nutritional composition of the host can also affect the growth and development of pests. Food plays a crucial role in influencing the nitrogen (N) and phosphorus (P) content within *C. davidi* larvae. As chestnuts increase in nitrogen (N) and phosphorus (P) content, the larvae also experience a significant increase in nitrogen (N) and phosphorus (P) content [31]. Temperature is a primary environmental variable that drives regional physiological adaptations by controlling metabolic rates, thereby altering the basic needs of organisms [32]. Therefore, we expected that temperature changes might affect the stoichiometry of diapause animals by impacting food composition or directly influencing the metabolic and storage requirements of the animals. Numerous studies have demonstrated that nitrogen (N) and phosphorus (P) concentrations decrease with rising temperatures, while carbon (C) concentrations remain relatively constant [33,34,35]. Carbon (C) primarily depends on temperature-related factors, indicating that it is mainly influenced by physiologically adapted temperatures. One possible explanation is that in warmer regions, *C. davidi* larvae contain higher carbon because metabolic demand is higher during diapause, causing the larvae to store more fat to meet their metabolic needs. The lower nitrogen (N) and phosphorus (P) content observed in larvae from warm regions is the result of the interaction between biological life strategies and climatic factors [36]. However, high temperatures have an accumulative effect on insect damage, and prolonged high-temperature conditions lead to a significant decrease in the water content of insects, ultimately causing their death [37].

The response curves plotted by MaxEnt software reflected the effect of a single environmental variable on the probability of the existence of *C. davidi*, ignoring the interaction between variables, which has some limitations. So, the conclusions obtained in this paper cannot fully elaborate on the role of environmental variables on *C. davidi* without considering other environmental factors affecting the distribution of *C. davidi*, such as host range, cultivation type, vegetation type, natural enemy distribution, etc., which will certainly lead to bias in the prediction results. In our future work, the influence of multiple compound environmental factors, including climatic factors, on the distribution of *C. davidi* will be considered comprehensively in an effort to make the prediction results more accurate.

## 5. Conclusions

*Curculio davidi* is a major pest in the chestnut-producing regions of China, but little research has focused on its potential distribution. Our work first utilized the MaxEnt model and ArcGis software to predict the potential distribution and trends of *Curculio davidi* under climate change. The prec7, bio18, bio4, bio15, bio10, and prec5 were identified as the key determinants affecting the distribution of *C. davidi*. In the current period, the highly adapted areas of *C. davidi*, 65,251 km^2^, accounting for 0.68% of the total area of China, were primarily concentrated in southwestern and eastern China. The moderately suitable areas under different future scenarios (SSP1-2.6, SSP2-4.5, and SSP5-8.5) increased by 22.12–27.33% (2050s) and 17.80–38.22% (2080s), respectively, compared to the current distribution, while the highly suitable areas showed a decreasing trend. This study provides data support for the management strategies of *C. davidi* and offers new insights into the dynamic changes of similar forestry pests.

## Figures and Tables

**Table 1:** The percent contribution and permutation importance of climatic variables in the MaxEnt modeling of *C. davidi*.

Variable	Percent Contribution (%)	Permutation Importance (%)
Precipitation from July (Prec7)	42.3	25.3
Precipitation of warmest quarter (Bio18)	22.3	3.4
Temperature seasonality (standard deviation × 100) (Bio4)	21.7	28.3
Precipitation Seasonality (Coefficient of Variation) (Bio15)	7.2	8.2
Mean Temperature of Warmest Quarter (Bio10)	4.1	28.9
Precipitation from May (Prec5)	2.4	5.8

**Table 2:** Analysis of main suitable distributions of *C. davidi*.

Province	High Suitable Area (km^2^)	Percentage of High Suitable Areas in Province (%)	Percentage of High Suitable Areas in China (%)
Gansu	68	0.02	0.10
Hebei	72	0.04	0.11
Shanxi	183	0.12	0.28
Yunnan	208	0.05	0.32
Shanghai	265	4.18	0.41
Guangxi	271	0.11	0.42
Liaoning	341	0.23	0.52
Guangdong	358	0.20	0.55
Fujian	899	0.74	1.38
Shanxi	1881	0.91	2.88
Zhejiang	3150	3.09	4.83
Shandong	3255	2.06	4.99
Chongqing	3650	4.43	5.59
Hunan	4887	2.31	7.49
Guizhou	5031	2.86	7.71
Jiangsu	5107	4.76	7.83
Jiangxi	5506	3.30	8.44
Anhui	6918	4.94	10.60
Sichuan	7256	1.49	11.12
Henan	7519	4.50	11.52
Hubei	8426	4.53	12.91
China	65,251	-	0.68

**Table 3:** Optimal ranges of environmental variables corresponding to the potential distribution of *C. davidi*.

Environmental Variables	Suitable Range	Optimum Value
Prec7/mm	107.22~324.13	103.13
Bio18/mm	305.02~1498.07	490.35
Bio10/°C	20.55~29.90; 29.90~39.95	23.58
Prec5/mm	44.08~299.32	55.15
Bio4/°C	674.2~1301.02	798.1
Bio15/mm	45.68~250.36	58.63

**Table 4:** Predicted suitable areas for *C. davidi* under current and future climatic conditions.

		Predicted Area (km^2^)			Comparison with Current Distribution (%)		
Decade	Scenarios	Poor Suitable Area	Moderate Suitable Area	High Suitable Area	Poor Suitable Area	Moderate Suitable Area	High Suitable Area
current		103,894	53,050	65,251			
2050 s	SSP1-2.6	102,064	64,784	56,843	−1.76	22.12	−12.89
	SSP2-4.5	111,728	67,547	56,868	7.54	27.33	−12.85
	SSP5-8.5	111,812	65,076	54,977	7.62	22.67	−15.75
2080 s	SSP1-2.6	97,362	62,492	55,799	−6.29	17.80	−14.49
	SSP2-4.5	102,121	73,324	45,859	−1.71	38.22	−29.72
	SSP5-8.5	119,996	64,867	47,705	15.5	22.28	−26.89

## Data Availability

*Curculio davidi* occurrence.xlsx. Figshare. Dataset (https://doi.org/10.6084/m9.figshare.26317339; accessed on 17 July 2024). The *C. mollissima* data supporting the results are available in a public repository: GBIF.org. GBIF Occurrence Download (https://doi.org/10.15468/dl.z6dhe5, accessed on 17 July 2024).
